# Anti-Inflammatory and Anti-Oxidant Properties of N-Acetylcysteine: A Fresh Perspective

**DOI:** 10.3390/jcm13144127

**Published:** 2024-07-15

**Authors:** Pierachille Santus, Juan Camilo Signorello, Fiammetta Danzo, Giada Lazzaroni, Marina Saad, Dejan Radovanovic

**Affiliations:** 1 Division of Respiratory Diseases, “L. Sacco” University Hospital, Università degli Studi di Milano, 20122 Milano, Italy; juan.signorello@unimi.it (J.C.S.); fiammetta.danzo@unimi.it (F.D.); giada.lazzaroni@unimi.it (G.L.); dejan.radovanovic@unimi.it (D.R.); 2 Department of Biomedical and Clinical Sciences, Università degli Studi di Milano, 20122 Milano, Italy; saad.marina@asst-fbf-sacco.it

**Keywords:** N-acetyl-L-cysteine, NAC, mucolytic, antioxidant, anti-inflammatory, NKA, IL-6, COPD, chronic respiratory disease

## Abstract

N-acetyl-L-cysteine (NAC) was initially introduced as a treatment for mucus reduction and widely used for chronic respiratory conditions associated with mucus overproduction. However, the mechanism of action for NAC extends beyond its mucolytic activity and is complex and multifaceted. Contrary to other mucoactive drugs, NAC has been found to exhibit antioxidant, anti-infective, and anti-inflammatory activity in pre-clinical and clinical reports. These properties have sparked interest in its potential for treating chronic lung diseases, including chronic obstructive pulmonary disease (COPD), bronchiectasis (BE), cystic fibrosis (CF), and idiopathic pulmonary fibrosis (IPF), which are associated with oxidative stress, increased levels of glutathione and inflammation. NAC’s anti-inflammatory activity is noteworthy, and it is not solely secondary to its antioxidant capabilities. In ex vivo models of COPD exacerbation, the anti-inflammatory effects have been observed even at very low doses, especially with prolonged treatment. The mechanism involves the inhibition of the activation of NF-kB and neurokinin A production, resulting in a reduction in interleukin-6 production, a cytokine abundantly present in the sputum and breath condensate of patients with COPD and correlates with the number of exacerbations. The unique combination of mucolytic, antioxidant, anti-infective, and anti-inflammatory properties positions NAC as a safe, cost-effective, and efficacious therapy for a plethora of respiratory conditions.

## 1. Introduction

N-acetyl-L-cysteine (NAC) was first introduced in 1965 primarily for its role in promoting mucolysis [1] and has been the preferred treatment for paracetamol intoxication since the mid-1970s [2]. Its intravenous formulation for this purpose is recognized on the WHO’s list of Essential Medicines [3].

The excessive production and secretion of airway mucus have significant importance in the pathophysiology and clinical manifestations of different respiratory diseases, including acute and chronic bronchitis (CB), chronic obstructive pulmonary disease (COPD), bronchiectasis, and cystic fibrosis (CF) [4,5].

Recent evidence indicates that oxidative stress and inflammation contribute to the development of these pathologies, showing a close correlation with clinical outcomes and triggering the overproduction of mucus by glands and goblet cells [6,7,8,9]. Under diverse stress conditions, such as infection, pathogenic factors, smoking, and oxidative stress, epithelial cells in the airway release pro-secretory molecules, leading to hypertrophy of secretory cells and hyperplasia of goblet cells, with concomitant overproduction of mucus [5,6,7,8].

Conditions like chronic bronchitis, bronchiectasis, CF, and COPD are associated with impairment of ciliary function and/or airway mucus hypersecretion [6,7,10,11].

COPD is characterized by airflow limitation, oxidative stress, and airway inflammation [12]. It is a persistent and advancing disease linked to a heightened inflammatory response in the respiratory tract triggered by gases, noxious particles, or cigarette smoke, which results in the injury of cells lining the airway and mucus hypersecretion [13]. In particular, the over-secretion of components of mucus MUC5AC and MUC5B in the respiratory tract, coupled with epithelial damage, leads to the shedding of ciliated cells, hyperplasia of goblet cells, and hypertrophy of the submucosal gland, ultimately resulting in airway mucus hypersecretion [4].

The mucus, secreted by serous, mucous, and goblet cells in response to various stimuli, including inflammation [9], becomes viscous [12] and promotes bacterial colonization and biofilm growth. This hinders the ability of neutrophils and soluble antimicrobial agents to permeate dense mucus and eradicate bacteria [14,15].

In COPD, numerous inflammatory mediators are synthesized and released in response to oxidative stress, such as the pro-inflammatory transcription factor NF-κB, which is over-expressed and activated by Reactive Oxygen Species (ROS), especially in airway epithelial cells and macrophages [7]. Oxidative stress also promotes the release of signaling molecules such as phosphoinositide 3-kinase (PI3K) and p38 mitogen-activated protein kinase (MAPK). It also sustains transforming growth factor (TGF)-β signaling and the expression of matrix metalloproteinase (MMP)-9, which are implicated in small airway fibrosis and emphysema [7]. Inflammation of the respiratory tract can contribute to flare-ups and deterioration of COPD [16]. Bronchiectasis involves irreversible bronchial lumen expansion, compromising mucus clearance [17] and facilitating bacterial colonization [18]. The initial production of toxins by bacteria contributes to the activation of both microbial and danger-associated molecular patterns, subsequently heightening inflammation [18]. As a result, this inflammatory response triggers hyperplasia and metaplasia of goblet cells, eventually resulting in increased secretion of mucus in the airways [19,20,21].

NAC has undergone extensive scrutiny in numerous global clinical trials and has been used for many years in treating various respiratory conditions [13,22,23]. It is a well-documented and well-established treatment of choice, valued not only for reducing mucus viscosity and promoting discharge but also for its recognized antioxidant and anti-inflammatory properties [13,22,23].

The aim of this review is to gather data on the multifaceted properties of NAC [13], with a specific focus on its anti-inflammatory role. The analysis emphasizes findings from various studies, highlighting the impact of dosage and treatment duration on NAC’s anti-inflammatory effectiveness [24,25,26,27,28,29]. The evidence suggests that lower doses may prove effective when administered over an extended treatment period [24,25,26,27,28,29]. The goal is to present NAC, traditionally recognized as a dual-action mucolytic and anti-oxidant agent, capable of fluidifying mucus secretions and clearing the airways, as a versatile drug with a multifaceted mechanism of action, rendering it a suitable pharmacological option for a variety of chronic respiratory pathologies characterized by mucus hyperproduction, oxidative stress, and inflammation.

## 2. Methods

The authors performed a comprehensive non-systematic review of the existing literature on PubMed/MEDLINE and Google Scholar, until February 2024, using the following keywords: “N-acetylcysteine” AND “anti-inflammatory” OR “mucolytic” OR “antioxidant” OR “chronic respiratory disease” OR “chronic obstructive pulmonary disease” OR “idiopathic pulmonary fibrosis” OR “bronchiectasis” OR “cystic fibrosis” OR “asthma” OR “bronchitis” OR “anti-infective”.

## 3. NAC Exhibits Pharmacological Actions beyond Mucolytic Activity

The mechanism of action for NAC is complex and multifaceted and is not yet fully elucidated. NAC is widely utilized to decrease the viscosity and elasticity of mucus [30]. It achieves this by breaking down the disulfide bonds in mucoproteins, thereby decreasing the viscosity of respiratory secretions [22].

Apart from the replenishing of depleted GSH content, facilitated by NAC, the gradual release of NAC-derived metabolites may result in a modest increase in H_2_S and its oxidation byproducts including persulfides (RSSH) and polysulfides (RSSnSR) [31], known as sulfane sulfur metabolites [32]. These metabolites exhibit cytoprotective effects by aiding in disulfide reduction, acting as direct scavengers of radicals, and preventing irreversible oxidative damage of proteins [32]. Moreover, modifications involving persulfide on proteins have the potential to modify their function and signaling capabilities, thereby initiating stress-responsive cellular reactions [32].

The initial use of NAC for respiratory diseases like chronic bronchitis and COPD is primarily linked to its direct and indirect mucolytic activity, aiming to reduce the viscosity of secretions and facilitate expectoration [22]. However, pre-clinical observations indicate that its effectiveness goes beyond its mucolytic properties, encompassing anti-oxidant, anti-infective, and anti-inflammatory effects. This unique combination of properties suggests an additional and beneficial impact on patients [13,22,33,34]. The diverse actions of NAC may have implications for reducing exacerbations in individuals with chronic inflammatory respiratory conditions, such as CB/COPD. NAC’s effectiveness has been thoroughly studied in this respiratory disease, and it is employed as an adjunctive medication endorsed by several internationally recognized guidelines [35,36,37,38]. Indeed, its therapeutic value and substantial supporting evidence have been acknowledged by both the American Thoracic Society and the European Respiratory Society guidelines [39].

### 3.1. NAC as Anti-Oxidant

Abnormal levels of GSH in both extracellular and intracellular compartments are commonly observed in chronic pulmonary diseases, such as CF, bronchiectasis, idiopathic pulmonary fibrosis (IPF), and COPD [40,41].

During the 1980s, Moldéus and colleagues [42] pioneered the revelation that NAC demonstrated anti-oxidant effects in respiratory cells and tissues. Their findings showcased NAC’s capability to safeguard against the detrimental impacts of cigarette smoke condensates and hydrogen peroxide (H_2_O_2_) [42]. The researchers proposed that NAC’s shielding effect against ROS may be attributed to its function as a precursor to GSH and an enhancer of GSH biosynthesis [42]. Indeed, from a stringent biochemical perspective, the anti-oxidant characteristics of NAC are intricately linked to the elevation of GSH content, leading to the reduction in ROS synthesis [22]. However, NAC also possesses the capability to directly engage with oxidants such as hydroxyl radical, hydrogen peroxide, and hypochloric acid [30,34].

The pharmacological characterization of NAC’s anti-oxidant effect took place in the human ex vivo model of acute exacerbations of COPD (AECOPD) consisting of lipopolysaccharide (LPS)-stimulated human bronchi. In two different studies performed by Cazzola et al. [27] and Calzetta et al. [28], NAC led to a decrease in LPS-induced malondialdehyde, H_2_O_2_, peroxidase activity, and nitric oxide (NO) production at concentrations of 1–10 µM and 16–35 µM, respectively. Notably, there was also a normalization of GSH levels in the aforementioned two separate studies [27,28], with one study reporting increased superoxide dismutase (SOD) activity by approximately 150% compared to LPS-treated bronchi [27].

Currently, oxidative stress is recognized as a key predisposing factor in the development of COPD, with an imbalanced oxidant/anti-oxidant state particularly pronounced during AECOPD [43]. Factors such as inflammation, microbial infection, or smoke exposure can increase GSH content in bronchoalveolar lavage fluid (BALF) as a protective measure against further lung damage [40]. While GSH concentration increases in the BALF of stable COPD patients, it diminishes during AECOPD [44]. This indicates that the increase in GSH levels within the lungs is inadequate to effectively counterbalance the significant production of ROS. Maintaining adequate GSH levels is crucial to counteract excessive ROS production during AECOPD [45]. Thus, therapeutic approaches focused on increasing GSH levels could potentially be beneficial in managing the severity of AECOPD and reducing the occurrence of future episodes.

In this context, NAC shows efficacy in restoring the depleted pool of intracellular GSH during AECOPD [46,47] by elevating GSH levels in plasma and BALF [48]. NAC also reduces ROS secretion by alveolar macrophages (AM) [49] and exhaled H_2_O_2_ and decreases H_2_O_2_ production in the respiratory tract of COPD patients [50]. This is corroborated by De Benedetto et al., who observed a reduction in exhaled H_2_O_2_ in patients with COPD after 2 weeks of treatment with NAC at 1200 mg/day administration [51]. Intriguingly, the reduction in exhaled H_2_O_2_ in patients with COPD who received 600 mg/day NAC was observed only after 9–12 months [50], indicating the need for a longer duration of treatment for anti-oxidant effect when lower doses are employed.

Collectively, the evidence presented strongly suggests that oxidative stress plays a pivotal role in the pathogenesis of lung abnormalities observed in individuals diagnosed with COPD. Alleviating oxidative stress is anticipated to lead to diminished pulmonary damage and lower the incidence of local infections, thereby significantly slowing the progression of COPD.

### 3.2. NAC as an Anti-Inflammatory Drug

The anti-inflammatory attributes of NAC have been substantiated through various studies [13]. In the 1980s, multiple investigations suggested that NAC could mitigate cigarette irregularities in polymorphonuclear neutrophils (PMNs) [52], AM [49,53,54], fibroblasts [42], and epithelial cells [55,56] induced by smoke.

In particular, when given to smokers at a high dosage over an 8-week period, NAC has demonstrated efficacy in diminishing airway inflammation by reducing plasma concentrations of elastase and myeloperoxidase, lowering the content of lactoferrin, anti-chymotrypsin, and eosinophil cationic protein (ECP) in BALF, and mitigating the chemotactic activity of neutrophils [57]. Furthermore, it enhanced AM function in smokers [49].

In the 1990s, Schreck et al. observed NAC’s effectiveness in reducing the activation of nuclear factor kappa B (NF-kB) in intact Jurkat T cells [58]. Further support for the anti-inflammatory effect of NAC was provided by data demonstrating a decrease in the chemiluminescence of PMNs in BALF in vitro [59]. Additionally, NAC administered 1 h before LPS exposure in rats showed a dose-dependent reduction in lung NF-κB activation, leading to significant suppression of endotoxin-induced neutrophilic alveolitis [60].

Cytokines are pivotal in pulmonary immune defense but may lead to lung injury when produced excessively or dysregulated [60]. Given NF-κB dependent regulation of various cytokines, it emerges as a key factor in the pathobiology of pulmonary injury caused by excessive inflammation [60]. Consequently, targeting the NF-κB pathway becomes an appealing strategy for therapies aiming to limit neutrophilic lung inflammation and host-derived lung injury [60]. NAC’s anti-inflammatory activity in cells of the respiratory tract was demonstrated by Desaki et al. [61] in 2000, showing its efficacy in reducing the activation of NF-κB in human bronchial epithelial cells treated with silica.

NAC orally administered at 600 mg/day has been reported to reduce sputum ECP and Interleukin-8 (IL-8) concentrations in patients with COPD [24,25]. Additionally, at this dosage, NAC modulated the inflammatory response after ten weeks of intake in COPD patients by decreasing serum IL-8 levels [26]. This intervention also removed the inverse relationship between the pro-inflammatory cytokine IL-8 and glutathione peroxidase (GPx) and Trolox equivalent antioxidant capacity (TEAC) [26]. These findings imply that NAC affects IL-8 expression through pathways that are not dependent on GPx and plasma anti-oxidants [26]. Moreover, it eliminated the positive correlation between intercellular adhesion molecule-1 (ICAM-1) and IL-8, indicating that ICAM-1 expression is modulated by pathways not susceptible to NAC [26].

A study examining how NAC treatment affects changes induced by sepsis in conscious rats revealed a reduction in inflammatory biomarkers (IL-6, tumor necrosis factor-α (TNF-α), and IL-10) linked to sepsis [62]. In vitro, NAC reduced inflammatory cytokines, namely IL-6, IL-1β, and TNF-α, in LPS-treated macrophages under mild oxidative conditions [63]. In a hamster model of lung damage induced by SARS-CoV-2, intravenous NAC at high doses (500 mg/kg) significantly decreased levels of pro-inflammatory cytokines such as IL-1β, IL-6, IFN-γ, and TNF-α [64]. As a result of this decrease, there was reduced infiltration of macrophages at the infection site, thereby preventing lung damage [64].

In particular, elevated IL-6 levels characterize chronic inflammatory conditions of the lung and have an active role in the development of chronic respiratory conditions such as COPD and asthma, designating it as a crucial pharmacological target [65]. Reflecting airway inflammation, IL-6 concentration is heightened in sputum [66] and exhaled breath condensate of COPD patients [67]. Correlating with the number of exacerbations, IL-6 elevation may be discerned in plasma/serum during exacerbations [66,68,69]. A recent study identified a serum IL-6 concentration of 14.030 pg/mL or higher as a risk factor for more than 2 AECOPD in the subsequent year [70].

Additional evidence indicates that IL-6 could be pivotal in exacerbating COPD, particularly in cases involving co-infection with both the Gram-negative pathogen *Haemophilus influenzae* and rhinovirus [71].

**Figure 1:**
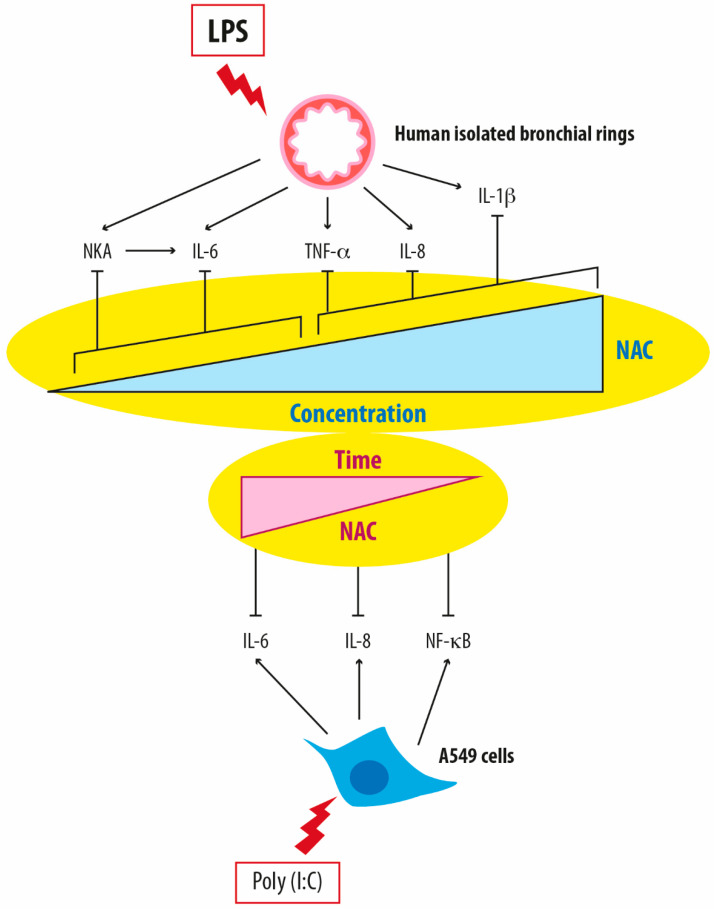
Differential modulation of pro-inflammatory mediators in relation to both the concentration and duration of treatment with N-acetylcysteine in in vitro models of airway inflammation. NAC: N-acetylcysteine; LPS: lipopolysaccharides; NKA: neurokinin A; IL-6: Interleukin-6; TNF-α: tumor necrosis factor-α; IL-8: Interleukin-8; IL-1β: Interleukin-1beta; NF-κB: Nuclear factor kappa B.

It has been recently demonstrated that NAC exerts both anti-oxidant and concentration-dependent effects in human-isolated bronchi of patients with COPD treated with LPS [27]. The aforementioned studies conducted by Cazzola et al. [27] and Calzetta et al. [28] explored the beneficial effect of NAC in reducing airway inflammation caused by LPS. They used a wide variety of concentrations applicable in vitro, including those mimicking the levels found in plasma following daily oral doses of NAC (200 mg, 600 mg, and 1200 mg) in a validated ex vivo model of AECOPD [27,28]. NAC has demonstrated efficacy in regulating neurokinin A (NKA) release in LPS-stimulated human bronchi at concentrations ranging from 1 to 10 µM [27] and 5 to 35 µM [28] in the two different studies (Figure 1). One of the two studies showed that higher NAC concentrations (300 μM to 10 mM) were needed to reduce the production of IL-8, IL-1β, and TNF-α from LPS-stimulated human bronchi by approximately 55% [27]. In contrast, lower concentrations (1 μM) sufficed to decrease IL-6 levels by approximately 33%, likely through the inhibition of NKA release [27] (Figure 1). Accordingly, the other study showed that selectively blocking the receptor for NKA NK2 nullified the association between the reduction in NKA release and the decrease in IL-6 content [28]. This finding suggests that NAC suppresses the neurogenic inflammatory response triggered by LPS by reducing NKA production, subsequently diminishing the increase in IL-6 [28].

Logistic regression analysis revealed that NAC’s influence on NKA levels correlated positively with IL-6 levels and reduced H_2_O_2_, malondialdehyde, peroxidase activity, and NO levels in both studies [27,28]. Notably, the study by Calzetta et al. [28] revealed that even very low concentrations, mirroring plasma levels from oral administration at 200 mg/day, were effective against GSH, H_2_O_2_, peroxidase activity, and IL-6. This effect was comparable in magnitude to that obtained with higher doses, reproducing the plasma levels resulting from oral administration at 1200 mg/day and 600 mg/day [28]. These findings indicate that NAC can modulate the production of NKA, leading to a subsequent beneficial effect against the LPS-induced increase in IL-6 when administered across a spectrum of doses (Figure 1).

A detailed, well-fitted logistic regression analysis of the results obtained by Calzetta et al. [28] demonstrated that the influence of NAC on NKA production displays a bell-shaped concentration-response curve. This suggests that the inhibitory effect observed at concentrations between 5 to 35 µM is greater than at higher concentrations [28]. This distinct fitting model induced by NAC concerning NKA levels may elucidate conflicting dose-effect findings reported in in vitro and in vivo settings, particularly regarding NAC’s anti-inflammatory activity [72].

These findings imply that additional mechanisms apart from the anti-oxidant activity control the rise of IL-6 in LPS-stimulated human bronchi, particularly when NAC is administered at low concentrations equivalent to an oral dose of 200 mg/day. This suggests a genuine anti-inflammatory effect of NAC, distinct from its anti-oxidant capabilities. Given that the LPS challenge triggers neurogenic inflammation in the airways and IL-6 exacerbates NKA release, NAC at low concentrations seems to finely regulate neurogenic inflammation [73,74,75,76], a harmful condition that contributes to the cyclical relationship between oxidative stress and inflammation in human bronchi [13,27].

Notably, a recent meta-analysis by Askari et al. [77] delving into the impact of oral NAC on serum inflammatory biomarkers uncovered that only a high dosage (≥1200 mg/day) of orally administered NAC effectively reduced C-reactive protein (CRP) levels. However, a noteworthy decrease in circulating IL-6 was reported with a dosage below 1200 mg/day [77]. This suggests that while high doses may be necessary for diminishing serum IL-6, these requirements are still lower than those needed for decreasing CRP levels. In their study [77], no discernible effect on the circulating levels of VCAM-1, ICAM-1, MCP-1, IL-8, and TNF-α was reported following oral administration of NAC. This aligns with the abovementioned findings of Cazzola et al. [27], where high concentrations of NAC (ranging from 300 μM to 10 mM) were required to reduce the release of IL-1β, IL-8, and TNF-α by LPS-stimulated bronchi.

Additionally, the duration of treatment significantly influences NAC’s anti-inflammatory activity, as evidenced by Montero et al.’s study [29]. Their findings showed that A549 cells treated with doses of NAC reflecting plasma concentrations after oral doses of 600 mg and 1200 mg prevented ROS induction, decreased thiol level, correlated with intracellular GSH levels, and suppressed IL-6 and IL-8 levels while inhibiting NFκB activation [29]. The extended incubation period enabled even lower concentrations of NAC to exhibit anti-inflammatory effects, suggesting that NAC’s positive impact on IL-6 regulation may stem from both its anti-oxidant properties and other anti-inflammatory mechanisms [27,28,29].

These results provide new evidence by confirming the concentration and time-dependent effects of NAC, both in vivo and in vitro, as suggested by Sadowska et al. [72]. NAC’s effects may be attributed to both its sustained anti-oxidant activity and its impact on intracellular events, including the regulation of NF-κB activation and NKA release.

## 4. Clinical Effectiveness of NAC in Pulmonary Medicine

**Figure 2:**
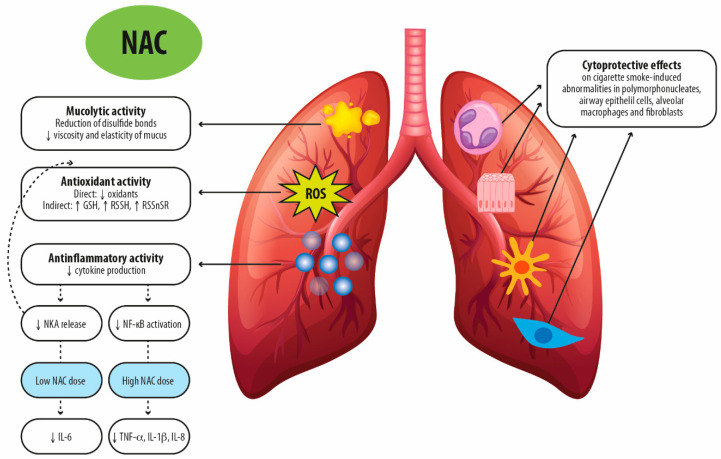
Proposed molecular mechanisms for the therapeutic activity of N-acetylcysteine after oral administration in chronic respiratory diseases. NAC: N-acetylcysteine; GSH: glutathione; RSSH: persulfides; RSS_n_SR: polysulfides; NKA: neurokinin A; NF-κB: Nuclear factor kappa B; TNF-α: tumor necrosis factor-α; IL-1β: Interleukin-1beta; IL-8: Interleukin-8; ROS: reactive oxygen species.

Pre-clinical observations on NAC suggest that its diverse pharmacologic characteristics, including mucolytic activity and specific anti-oxidant and anti-inflammatory effects, may have a beneficial impact on COPD (Figure 2).

A correlation between exacerbation frequency and sputum IL-8 and IL-6 levels has been established [68]. NAC, by acting on baseline levels of these cytokines, may contribute to limiting the inflammatory status and modulating exacerbation frequency. Additionally, by reducing sputum viscosity, NAC may facilitate airway clearance, influencing the course of exacerbation, as there is a link between bacterial colonization and exacerbation frequency and character [78].

The anti-infective properties and the ability to modulate the human bronchial tone of NAC can also impact the progression of COPD [13]. Patients experiencing frequent AECOPD may exhibit airway colonization by bacteria that produce biofilm [79], potentially perpetuating a harmful cycle of infection and inflammation that contributes to disease progression [80,81,82]. NAC’s antimicrobial activity has been demonstrated against various microorganisms, affecting different steps of biofilm formation [33,79]. NAC may also demonstrate strong anti-mycobacterial effects, reducing *Mycobacterium tuberculosis* infection and disease [83] and enhancing the effectiveness of antibiotics against bacterial infections [84,85,86,87,88]. However, it’s crucial to highlight that the plasma concentrations of NAC needed for its anti-infective properties can be much higher than what can be achieved through oral administration [13].

NAC has been shown to counteract bronchial desensitization during LPS challenges, restoring the normal contractility of airway smooth cells in an ex vivo AECOPD model [27], suggesting a potential role in protecting against exacerbations.

While certain dated studies found a small but significant positive impact of NAC on respiratory function in stable COPD patients, as measured by FEV_1_ [89,90] and MEF_50_ [90], recent research yielded disappointing results [91,92,93]. This discrepancy may stem from the limited sensitivity of these measures in detecting small airway disease and air trapping, where NAC is believed to exert its greatest effect, as evidenced by the improved FEF_25–75%_ observed in the BRONCUS trial [91].

The randomized placebo-controlled HIACE and PANTHEON trials highlight a reduction in exacerbation rates in patients with COPD treated with 1200 mg/day of NAC for one year [88,94]. This effect appears more pronounced in stable COPD patients and mild disease cases, irrespective of the AECOPD rate [88,94]. Accordingly, a meta-analysis by Shen et al. [95] suggests that extended therapy with high doses of NAC (>600 mg/day) can decrease AECOPD.

Another meta-analysis conducted by Cazzola et al. [96] reports that oral administration of NAC for 4 months or more effectively reduces the frequency of AECOPD. However, higher doses (≥1200 mg/day) were needed for this effect, while NAC at the dosage of ≤600 mg/day prevented exacerbation of chronic bronchitis [96]. In another meta-analysis, the same authors also highlight that high doses of NAC (1200 mg/day), employed as additional therapy in COPD patients, prevent AECOPD [97].

In contrast, a systematic review by Fowdar et al. [92] indicates that both low-dose NAC (≤600 mg/day) and high-dose NAC (>600 mg/day) can safely reduce the percentage of patients experiencing one exacerbation or more after at least 6 months of treatment. This suggests that administering NAC over the long term can decrease the likelihood of COPD exacerbations, irrespective of dosage. In chronic bronchitis, NAC 600 mg/day over a duration of 3–6 months prevented acute exacerbations and improved symptoms [98,99].

A recent meta-analysis including twenty studies and a total of 4044 patients evaluated oral NAC efficacy either in patients with CB/pre-COPD (patients with chronic bronchitis and no diagnosis of COPD) or with confirmed COPD: in both groups, patients treated with NAC exhibited a noteworthy decrease in the occurrence of exacerbations [100]. Furthermore, CB/pre-COPD patients treated with NAC showed a significant likelihood of experiencing symptom improvement and/or improved QoL compared to those receiving a placebo [100].

NAC is linked to a favorable safety profile, with a beneficial impact observed in both patients with stable COPD and individuals at risk of AECOPD, regardless of inhaled corticosteroid use [88,92,96,97,101].

Some years ago, mucolytic agents, like NAC, were recognized as the most cost-effective treatment option for managing severe COPD patients prone to frequent exacerbations [102].

Based on the currently available findings, the Global Initiative for Chronic Obstructive Lung Disease (GOLD) strategy incorporates a section titled “Mucolytic (mucokinetics, mucoregulators) and anti-oxidant agents (NAC, carbocysteine)” [35]. The recommendations explicitly state that “in COPD patients not receiving inhaled corticosteroids, regular treatment with mucolytics such as carbocysteine and N-acetylcysteine may reduce exacerbations and modestly improve health status” [35]. Accordingly, the European Respiratory Society/American Thoracic Society guideline emphasizes that mucolytic therapy diminishes the probability of hospitalization in COPD patients [39]. Moreover, when administered in elevated doses, it has the potential to decrease exacerbations [39].

Extensive evidence from randomized clinical trials and thorough meta-analyses supports the efficacy of long-term NAC administration in decreasing the risk of AECOPD [88,94,96,97] and establishes a dose-related protective effect [96,97]. Additionally, pre-clinical in vitro studies indicate that chronic administration may require lower doses for efficacy [97]. However, there remains a crucial need for larger and more robust randomized controlled trials to optimize dosage and duration of treatment. Such trials can contribute to instilling greater confidence and facilitate the widespread adoption of NAC as a safe and effective therapy for COPD management.

Concerning lung diseases other than COPD, thiol drugs have been proposed to have anti-fibrotic activity in IPF, attributed to their antioxidant and anti-inflammatory effects [103,104,105]. This condition involves the generation of oxygen radicals and a reduction in GSH levels [106]. Despite these potential benefits, clinical trials for IPF have produced inconsistent results [103,106,107,108]. Using oral NAC, either by itself or in conjunction with pirfenidone or nintedanib, might be an effective treatment option for certain IPF patients [109], particularly those with the rs3750920 (TOLLIP) TT genotype [110]. Data on the overall efficacy and safety of NAC in a pharmacogenomic context, tailored to patients most likely to benefit, are eagerly awaited [108].

In addition, NAC is also utilized for treating non-CF bronchiectasis, where it could help decrease the frequency of exacerbations [111]. A recent randomized placebo-controlled pilot study evaluated the impact of NAC on sputum neutrophil elastase levels, which serve as a surrogate marker for exacerbations, in adult patients with bronchiectasis [112]. They observed a nearly 50% reduction in sputum neutrophil elastase and improvements in lung function and QoL-related domains, without increased adverse events compared to placebo [112].

A multicenter, double-blind, randomized, placebo-controlled trial is presently in progress to investigate the sustained effectiveness of oral NAC on exacerbation rates and QoL in bronchiectasis [113]. The study aims to specifically focus on the antioxidant, anti-inflammatory, and antibacterial properties of NAC in bronchiectasis [113].

NAC is also used in CF, which is characterized by frequent respiratory infections, PMN-mediated inflammation, and increased oxidative stress [114]. However, evidence of NAC effectiveness in CF is still poor, despite some encouraging results [115,116,117], and further studies on larger samples are needed [114].

Interestingly, NAC as adjunctive therapy has been associated with reduced rates of progression to respiratory failure, with some studies reporting shorter hospital stays and others indicating decreased mortality in patients hospitalized for COVID-19 pneumonia [118].

## 5. Conclusions

In conclusion, although adjusting the anti-oxidant status may indirectly trigger anti-inflammatory effects, recent findings in vitro [28] highlight a distinctive anti-inflammatory activity of NAC at low concentrations, equivalent to an oral dosage as low as 200 mg/day. This bolsters the evidence suggesting that NAC may disrupt the harmful cycle between oxidative stress and inflammation, a detrimental condition prevalent in the airways of patients undergoing acute exacerbations of chronic respiratory conditions [13]. The prospect of utilizing NAC in COPD treatment appears promising, especially with prolonged administration (beyond 6 months). While the proposed mechanism, potentially involving NF-κB inhibition and the inhibition of NKA release, holds promise for influencing the production of diverse mediators and regulating inflammation more profoundly, further investigation is required for a comprehensive understanding. Clinical trial results suggest that prolonged NAC administration can significantly enhance respiratory symptoms and decrease the frequency of exacerbations in COPD and CB/pre-COPD patients.

## Data Availability

The raw data supporting the conclusions of this article will be made available by the authors upon request.
